# Development of Vancomycin, a Glycopeptide Antibiotic, in a Suitable Nanoform for Oral Delivery

**DOI:** 10.3390/molecules30071624

**Published:** 2025-04-05

**Authors:** Ali A. Amer, Lewis Bingle, Cheng Shu Chaw, Amal Ali Elkordy

**Affiliations:** 1 School of Pharmacy and Pharmaceutical Sciences, Faculty of Health Sciences and Wellbeing, University of Sunderland, Sunderland SR1 3SD, UK; bh77lq@student.sunderland.ac.uk (A.A.A.); hs0cch@sunderland.ac.uk (C.S.C.); 2 School of Nursing and Health Sciences, Faculty of Health Sciences and Wellbeing, University of Sunderland, Sunderland SR1 3SD, UK; lewis.bingle@sunderland.ac.uk

**Keywords:** vancomycin, niosomes, fast-disintegrating film, antimicrobial test, Span 60

## Abstract

Bacterial infections caused by resistant strains have emerged as one of the most significant life-threatening challenges. Developing alternatives to conventional antibiotic formulations is crucial to overcoming these challenges. Vancomycin HCl (VCM) is a glycopeptide antibiotic used for Gram-positive bacterial infections that must be given intravenously for systemic infections since it cannot pass through the gut wall due to its chemical structure and characteristics. The aim of this research is to develop VCM in a niosomal nanoform to then be encapsulated in fast-disintegrating oral films for effective delivery to enhance the application of vancomycin-loaded niosomes for treating oral infections and to be used in dental treatments. The formulation of niosomes encapsulating VCM was conducted with various ratios of Span 40, Span 60, and cholesterol as well as Kolliphor RH40 and Kolliphor ELP as co-surfactants using the microfluidic technique. The prepared niosomes were characterised using dynamic light scattering (DLS) for their size determination; high-pressure liquid chromatography, HPLC, for drug encapsulation efficiency determination; and the agar diffusion method for the determination of the antibacterial efficacy of the VCM niosomes against *Bacillus subtilis*. The niosomal formulation was then incorporated into polyvinyl alcohol (PVA) film, and the properties of the oral film were characterised by in vitro assays. The vancomycin-loaded niosomes produced with optimal conditions exhibited small diameter with acceptable polydispersity index, and drug encapsulation efficiency. This study presents multifunctional niosomes loaded with VCM, which demonstrated efficient in vitro activity against Gram-positive bacteria upon the slow release of VCM from niosomes, as demonstrated by the dissolution test. Oral films containing VCM niosomes demonstrated uniform weights and excellent flexibility with high foldability and a rapid disintegration time of 105 ± 12 s to release the niosomal content. This study showed that the microfluidic approach could encapsulate VCM, a peptide in salt form, in surfactant-based niosomal vesicles with a narrow size distribution. The incorporation of niosomes into fast-disintegrating film provides a non-invasive and patient-friendly alternative for treating bacterial infections in the oral cavity, making it a promising approach for dental and systemic applications.

## 1. Introduction

**Figure 1:**
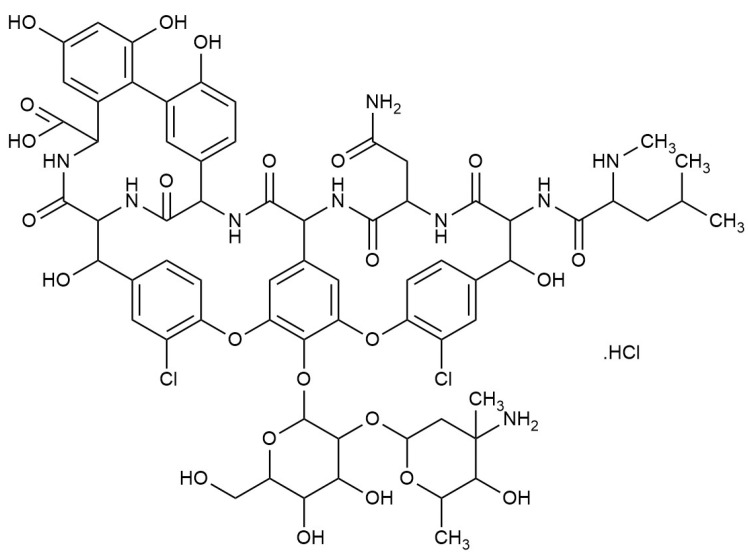
Vancomycin hydrochloride structure (Drawn by ChemDraw).

**Figure 2:**
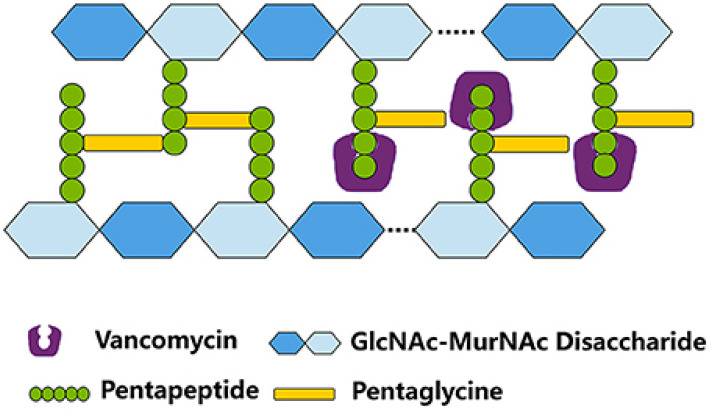
A schematic representation of the binding of VCM to the D-Ala-D-Ala terminus of the pentapeptide, blocking transglycosylation and the subsequent cross-linking of peptidoglycan chains [11].

Improving drug permeability and absorption is one of the biggest problems facing the pharmaceutical industry [1]. The size of the particles is one of the most important parameters influencing medication permeability. Accordingly, antibiotic nanoparticles have exceptional antibacterial effectiveness and improved tissue penetration [2]. Vancomycin (VCM) is a well-known glycopeptide antibiotic (Figure 1) which is used to treat Gram-positive bacterial infections, and it works quite effectively against Gram-positive bacteria [3]. It is also crucial for treating infections brought on by bacteria that are resistant to antibiotics, such as methicillin-resistant *Staphylococcus aureus* (MRSA), which is resistant to both penicillin and methicillin [4]. VCM can be a recommended course of treatment for patients who are hypersensitive to beta-lactam antibiotics since it has a potent inhibitory impact against *Staphylococcus* and *Streptococcus* species [5]. VCM must be given intravenously for systemic infections since it cannot pass through the gut wall. This antibiotic is often used to treat complex or severe infections such as meningitis, bloodstream infections, endocarditis, or bone and joint infections [6]. Monitoring plasma concentrations is crucial for accurate dosing in these circumstances. VCM works by preventing Gram-positive bacteria from synthesising their cell walls; it is mostly ineffective against Gram-negative bacteria. It inhibits the development of bacterial cell walls by binding with high affinity to the D-Ala-D-Ala terminus of the pentapeptide, blocking transglycosylation and the subsequent cross-linking of peptidoglycan chains [7]. Figure 2 shows a schematic representation of the binding of VCM to the D-Ala-D-Ala terminus of the pentapeptide, blocking transglycosylation and the subsequent cross-linking of peptidoglycan chains. Additionally, VCM is a large hydrophilic molecule; it has poor gastrointestinal absorption and is mainly used in this form to target *Clostridium difficile* colitis in the large intestine [8]. The development of VCM formulations based on nanoparticles offers a promising way to overcome the limitations of oral administration, which currently limits its use for systemic infections. Oral administration is often the preferred route due to its simplicity and improved patient compliance, but in the case of VCM, this method is severely limited by its poor absorption profile [9]. After intravenous administration, VCM extensively distributes across various tissues. It has a distribution volume of approximately 50 litres (Vd = 0.4 L/Kg), with a protein-binding rate ranging between 37% and 55% [10]. The elimination of VCM primarily occurs through the renal route, and it can be detected in multiple bodily fluids, including cerebrospinal fluid (in cases of inflamed meninges), bile, pericardial fluid, and synovial fluid [6]. When administered orally, drug absorption necessitates the passage of molecules through one or more biological membranes before entering the bloodstream. The ability of a drug to pass through these membranes is known as permeability, which is a crucial biopharmaceutical parameter that dictates its absorption, distribution, metabolism, and excretion. Nanoencapsulation has been investigated for targeted therapy, controlled drug release, minimising side effects, increasing therapeutic performance, preserving steady drug concentrations in tissues and blood, and boosting overall treatment efficacy [2]. The main goals of oral nanoparticle administration are to improve bioavailability, control release, and minimise gastrointestinal discomfort brought on by the medication.

Niosomes have recently been successfully used to not only encapsulate small drug molecules [12] but also to encapsulate macromolecules, for instance albumin and insulin, with positive outcomes using conventional thin-film hydration and reversed-phase techniques, respectively [13,14].

**Figure 3:**
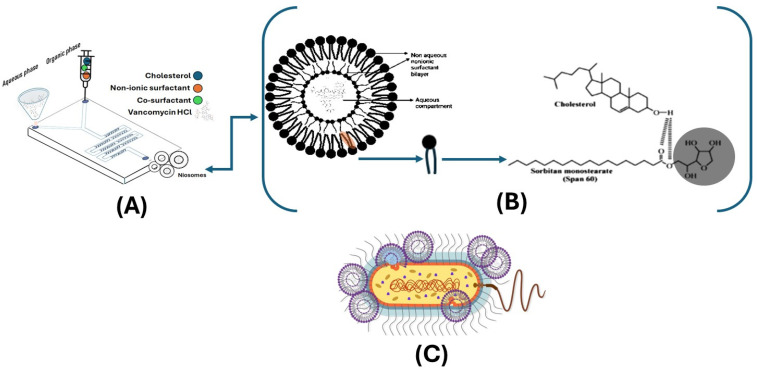
Microfluidic technology for niosome synthesis (**A**), niosome structure with the integration of the non-ionic surfactant Span 60 with cholesterol (**B**), and explanatory antimicrobial action of vancomycin-loaded niosomes (**C**).

Structurally, niosomes are formed by the self-assembly of non-ionic surfactants, typically comprising alkyl or dialkyl polyglycerol ethers, in combination with cholesterol. This assembly results in the formation of closed bilayer vesicles, wherein the hydrophobic tails of the surfactants align inward to create the bilayer, while the hydrophilic heads orient outward, forming a hydrophilic core (Figure 3B). This bilayer structure enables niosomes to encapsulate hydrophilic drugs within the aqueous core and hydrophobic drugs within the bilayer space, offering a highly adaptable platform for drug delivery [15]. The physicochemical characteristics of niosomes, including their size and lamellarity (number of bilayer membranes), are influenced by both intrinsic factors, such as the non-ionic surfactant concentration, chemical structure, non-ionic surfactant interactions, and the balance of repulsive and attractive forces, and extrinsic factors, like the method of preparation, drug hydrophobicity, surfactant-to-drug ratio, and molecular weight of the encapsulated drug. These parameters can be finely tuned to optimise niosome formulations for specific therapeutic applications, including achieving controlled release profiles, enhancing stability, and improving target specificity [16]. Additionally, niosomes can be engineered for targeted delivery, thereby reducing off-target effects and improving drug localisation at the intended site of action [17].

Microfluidic technology is an advanced method for the precise fabrication of nanoparticles, including lipid-based structures like liposomes and cationic lipid nanoparticles, and its applications for the entrapment of soluble peptide agents in surfactant-based niosomal formulations remain sparse. By manipulating fluids at the microscale, this technique achieves rapid mixing and the efficient encapsulation of active compounds. In a typical microfluidic setup, an aqueous phase containing the active drug or compound and an organic phase with dissolved non-ionic surfactants are forced to converge within microchannels (Figure 3A). This controlled environment allows for the formation of nanoparticles with uniform size and morphology, offering several advantages over traditional methods, such as enhanced control over particle size, narrow size distributions, and high reproducibility. Moreover, microfluidics enables scalable production while maintaining consistent quality, making it particularly valuable for pharmaceutical applications [18].

In the treatment of bacterial infections, niosomes have shown considerable promise as carriers for antibiotics [19]. By encapsulating antibiotics, niosomes can enhance the penetration of drugs into bacterial cells, improve their stability, and achieve sustained release, thereby increasing the efficacy of the treatment and reducing the risk of antibiotic resistance (Figure 3C) [20].

In order to produce a pharmacodynamic response, medicines must successfully penetrate membranes and reach their target site in adequate concentrations. Drugs administered via the buccal route must first pass through the mucous layer before diffusing into the buccal epithelium and being absorbed [21,22]. The oral mucosa often absorbs small, lipophilic medications (log *p* 1.6–3.3) well, but drugs with larger log *p* values exhibit a lower absorption rate because of their restricted water solubility [23]. The oral mucosa is mostly penetrated by lipophilic small molecules via the transcellular pathway. On the other hand, big hydrophilic molecules have a harder time entering the oral mucosa, which includes the non-keratinised buccal and sublingual tissues. Their absorption usually occurs through the paracellular route, which makes use of the amphiphilic qualities of intercellular lipids. Moreover, salivary pH can affect the charge and hydrophilic or hydrophobic characteristics of molecules, potentially impeding their absorption [24].

Nanoparticles are effective drugs carriers that minimise the limitations of buccal drug delivery. These nanocarriers increase the drug’s passage across the mucous layer, protect it against deterioration, and reduce dilution in saliva, among other advantages. Nanoparticles improve the duration of residence and interaction with the ability of attaching to the buccal mucosa layer [25]; niosomes are a well-known nanoparticulate system for targeted drug delivery [26]. The salivary film that covers the mucosal surface facilitates the absorption of hydrophilic molecules while acting as a barrier to the penetration of lipophilic substances. The aqueous character of saliva is thought to be the cause of this selective permeability, which makes hydrophilic polymer-based nanoparticles more advantageous for efficient penetration [27]. Because mucus contains negatively charged sialic acid, neutral or positively charged nanoparticles show improved mucoadhesion. Higher localised medication distribution is made possible by this intimate interaction with the mucus, which also lengthens the retention period. However, the absorption of carriers, especially those with lipophilic qualities, is restricted by the mucosal cells and normal turnover. Diffusion kinetics are also influenced by mucus structure organisation and nanoparticle size [28]. Saliva’s pH is crucial for the regulated release of drugs from nanocarriers. Although the permeability of drugs that ionise in acidic conditions is decreased, their penetration efficiency can be improved by employing techniques that increase the percentage of non-ionised drug molecules [27]. Drug-loaded nanoparticles can be absorbed through the buccal epithelium in two main ways: the transcellular route, in which the drug passes straight through epithelial cells, and the paracellular route, in which it moves through the gaps between neighbouring epithelial cells [29].

The primary aim of this study is to develop and characterise vancomycin hydrochloride-loaded niosomes using microfluidic techniques to determine their effectiveness in producing niosomes with optimal properties. This study will investigate the impact of various excipients, including Span 60, Span 40, Kolliphor RH40, and Kolliphor ELP, on the size, shape, and encapsulation efficiency of the vancomycin-loaded niosomes. By using different non-ionic surfactants and cholesterol ratios, this study seeks to identify the most effective surfactant combinations for niosome formulations. Additionally, factors such as drug loading and surfactant concentrations and flow rate ratios will be examined to assess their influences on niosome properties. Beyond formulation, this study will evaluate the in vitro release profiles of vancomycin-loaded niosomes and assess their antibacterial efficacy against Gram-positive bacterial strains to determine their therapeutic potential. The second aim of this work will focus on integrating vancomycin-loaded niosomes into a fast-disintegrating oral film. This innovative formulation is designed to treat systemic infections and other dental conditions, ensuring efficient drug delivery. Vancomycin-loaded niosome fast-dissolving oral films (FDOFs) will be evaluated for key physicochemical parameters such as weight uniformity, film thickness, flexibility, tensile strength, and surface pH to ensure batch-to-batch consistency and quality. By optimising both the film formulation and niosome characteristics, this study aims to develop a stable and effective niosomal drug delivery system with enhanced therapeutic benefits.

## 2. Materials and Methods

### 2.1. Materials

Vancomycin hydrochloride was purchased from the Molekula Group (Darlington, UK); Span 60 (sorbitan monostearate), Span 40 (sorbitan monopalmitate), cholesterol, and polyvinyl alcohol (PVA), Mw 13,000 Da, were purchased from Merck (Darmstadt, Germany). Kolliphor RH40 (polyoxyl 40 hydrogenated castor oil), Kolliphor ELP (polyoxyl 35 castor oil), and polyethylene glycol 400 (PEG400) were purchased from BASF (Stockport, UK). Dialysis membranes (MWCO 3 KDa) were purchased from MEDICALL Ltd. (Oldham, UK). The standard Gram-positive bacteria *Bacillus subtilis* NCTC 3610 and all culture media were provided by the microbiological collection bank of the Microbiology Laboratory, University of Sunderland. All chemicals were pharmaceutical-grade with high purity.

### 2.2. Methods

#### 2.2.1. Niosome Formulations

Five different niosomal formulations were developed using varying molar ratios of cholesterol (Ch), Span 60 (SP60), Span 40 (SP40), Kolliphor RH40 (RH40), and Kolliphor ELP (ELP) to assess their influence on vesicle characteristics. The total mass of each formulation varied depending on the molecular weights of the individual components, ranging between 120 mg and 140 mg for a combined 150 µmol of components. To standardise the formulations for comparison, each was scaled to a total mass of 40 mg by applying an appropriate scaling factor based on its initial total mass, ensuring that the molar ratios remained consistent (Table 1). The choice of the molar ratio of cholesterol and non-ionic surfactants was dependent on different experiments that were performed on methylene blue as a hydrophilic model drug where the non-ionic surfactant and co-surfactant ratio remained constant to examine the impact of using different compartments on niosome physicochemical properties. All formulations were prepared in duplicate (n = 2) and tested in triplicate (n = 3) to ensure the reproducibility and accuracy of the results.

#### 2.2.2. Microfluidic Method

Niosomes containing vancomycin hydrochloride were prepared using the microfluidic technique (Precision NanoAssemblr™ Benchtop, Vancouver, BC, Canada). The required amounts of Span 60, Span 40, Kolliphor RH40, and Kolliphor ELP were accurately weighed. These surfactants were dissolved in 1 mL ethanol as an organic solvent to create a homogeneous surfactant solution. The aqueous phases containing vancomycin hydrochloride at the desired concentrations were dissolved in 3 mL Trizma buffer, pH 7.4. Both solutions were injected into the microfluidic device through separate inlets, where they were mixed in a controlled manner within the device’s mixing chamber, as shown in Figure 3A. This precise mixing promoted the spontaneous formation of niosomes, encapsulating the drug within the niosome vesicles. The microfluidic device was set up with a total flow rate (TFR) of 12 mL/min, maintaining aqueous-to-organic phase ratios of 3:2 and 3:1 as the flow rate ratio (FRR) and 60 °C for all formulations. The formed niosomes were then collected and stored at 4 °C to maintain stability.

#### 2.2.3. Niosome Characterisations

The characterisation of niosomes was performed to assess their size, morphology, and encapsulation efficiency (EE). The vesicle size and polydispersity index (PDI) of the size distribution were determined using a Zeta-sizer device (Malvern Instrument Ltd., Malvern, UK), where 50 times dilution of the sample that contains the niosomal suspension was analysed at room temperature. The average hydrodynamic diameter was recorded, providing insights into the size and uniformity of the niosomes. For morphological analysis, light microscopy (Bioblue Eurmax, Rotterdam, The Netherlands) was used to observe surface characteristics regarding the shape of the niosomes.

The niosomes were initially prepared using microfluidic techniques, resulting in a suspension containing both encapsulated and unencapsulated drug molecules. The encapsulation efficiency (EE) was evaluated by first separating the unencapsulated vancomycin hydrochloride through centrifugation. To purify the niosomes, centrifugation was performed at a speed of 15,000 rpm for 60 min at a controlled temperature of 2–4 °C to prevent vesicle degradation using a microcentrifuge (Hettich MIKRO 200R, Kirchlengern, Germany). The clear supernatant solutions were collected and quantified using HPLC, although the amount of encapsulated drug was measured to confirm that the amount of the drug in the supernatant matched with the amount in the pellet. The encapsulation efficiency was calculated according to the equation below, Equation (1), by comparing the amount of unencapsulated drug to the total drug initially added, providing a robust measurement of the formulation’s effectiveness in drug incorporation. These characterisation techniques ensure that the niosomes meet the desired criteria for size, morphology, and drug loading.

(1) EE%=((Total drug−Free drug)/Total drug)×100

#### 2.2.4. HPLC Calibration Curve Method

**Figure 4:**
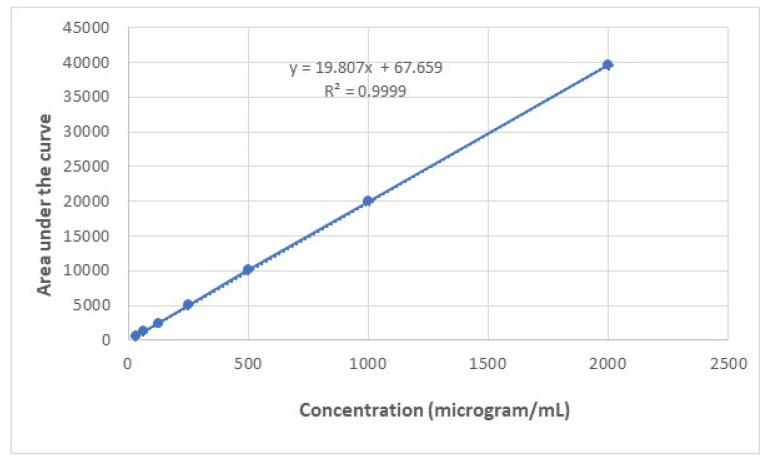
Calibration plot for vancomycin hydrochloride measured Using HPLC. Data represent mean ± standard deviation (n = 3).

To develop a calibration curve for vancomycin hydrochloride using High-Performance Liquid Chromatography (HPLC, Agilent series 1100, Santa Clara, CA, USA), a series of standards was prepared at concentrations of 31.25, 62.5, 125, 250, 500, 1000, and 2000 µg/mL. The HPLC system was equipped with a C18 analytical column (100 mm × 4.6 mm, 5 µm particle size) and operated with a mobile phase composed of water containing 0.1% formic acid and 20 mM ammonium format (Mobile Phase A) and methanol containing 0.1% formic acid and 20 mM ammonium format (Mobile Phase B). A gradient elution was used, starting with 100% A and 0% B, linearly changing to 0% A and 100% B over 5 min, then holding 100% B for 1 min, before returning to the initial conditions and re-equilibrating. The flow rate was set at 1.0 mL/min, the column temperature was maintained at 28 °C, and the detection was performed at 230 nm. Calibration standards were prepared by dissolving vancomycin hydrochloride in Trizma buffer, pH 7.4, to make a stock solution of 2000 µg/mL, followed by serial dilutions to achieve the desired concentrations. A 20 µL aliquot of each standard was injected, and the peak areas were recorded at 3.154 min, which was the retention time. The calibration curve was constructed by plotting the peak areas against the corresponding concentrations, and the linearity of the curve was assessed by calculating the correlation coefficient (R), as shown in (Figure 4). Data are presented as the mean ± SD (n = 3).

#### 2.2.5. In Vitro Drug Release

The in vitro release study of vancomycin-loaded niosomes was conducted using the dialysis bag method to compare three different formulations (M1, M2, M3—prepared with a flow rate ratio of 3:2, at 1 mg/mL drug loading and 20 mg/mL surfactant concentration, and a 1 mg/mL free-drug solution as a control; hence, all samples have the same drug concentration. Each formulation was placed in a dialysis bag with a 3000 Da molecular weight cut-off to retain the niosomes while allowing the free drug to diffuse out. The dialysis bags were then immersed in 50 mL of Trizma buffer (pH 7.4) to maintain a mouth environment with continuous stirring using Magnetic Hotplate Stirrers (Fisher Scientific, Leicestershire, UK) at 37 °C and 50 RPM. Then, 1 mL samples were collected at 0, 0.5, 1, 2, 3, 5, 6, 12, 24 and 30 h time intervals. After each sampling, 1 mL of fresh Trizma buffer was added to maintain a constant volume. The samples were analysed for VCM content using the HPLC method described in Section 2.2.4, to determine the concentration of the drug released at each time point. The cumulative percentage of VCM released was calculated for each formulation, and the release profiles were compared against the control to evaluate the drug release. Triplicates were analysed for the sample batch. This study provided insights into the sustained release characteristics of the niosomal formulations and helped identify the most optimised formulation.

#### 2.2.6. Antimicrobial Activity

To assess the antibacterial activity of vancomycin-loaded niosomes, an antibiotic susceptibility assay was conducted using the agar diffusion method against the Gram-positive bacterium *Bacillus subtilis* [30] and the Gram-negative bacterium *Escherichia coli*. The bacterial culture was initially cultivated in nutrient broth and incubated overnight at 37 °C to achieve the stationary phase. The bacterial suspension was subsequently transferred to a sterile saline solution and mixed thoroughly using a vortex mixer. The turbidity of the suspension was then adjusted to a density of 0.5 McFarland units using a densitometer, bio-DEN-1 (Grant, Swindon, UK). The surface of a nutrient agar plate was uniformly inoculated with the bacteria culture using a sterile cotton swab to ensure consistent bacterial distribution.

Three VCM-loaded niosome formulations (M1, M2, M3) were centrifuged to remove the unentrapped VCM. The pellets were reconstituted and centrifuged 2 times with fresh Trizma buffer, pH 7.4, to remove any vancomycin residues. The pellets were then dispersed on paper disks in an allocated volume between 5 and 20 µL. As controls, free VCM hydrochloride, 1 mg/mL, and blank niosomes (without the drug) were similarly prepared at a 20 mg surfactant/mL concentration. All samples had the same concentration of VCM, 1 mg/mL. Sterile paper discs (6 mm in diameter) were then soaked with the VCM-loaded niosome suspension, free-VCM solution, and blank niosomes. These discs were placed on the inoculated agar plates. The plates were left at room temperature for 15–20 min to allow for the diffusion of the drug from the discs into the agar medium. Subsequently, the plates were incubated at 37 °C for 24 h to allow bacterial growth and the formation of inhibition zones.

After incubation, the clear areas around the discs where bacterial growth was inhibited were carefully measured using a ruler. The diameter of each zone was recorded in millimetres, providing a quantitative measure of the antibacterial activity. The effectiveness of the vancomycin-loaded niosomes was assessed by comparing the size of these inhibition zones with those produced by free vancomycin and blank niosomes. Larger zones of inhibition indicated greater antibacterial efficacy, demonstrating the potential of niosomal formulations for enhanced drug delivery. This optimised agar diffusion method allows for an evaluation of the antibacterial potency of vancomycin-loaded niosomes against *Bacillus subtilis* as a Gram-positive model.

#### 2.2.7. Oral Film Preparation Method

**Figure 5:**
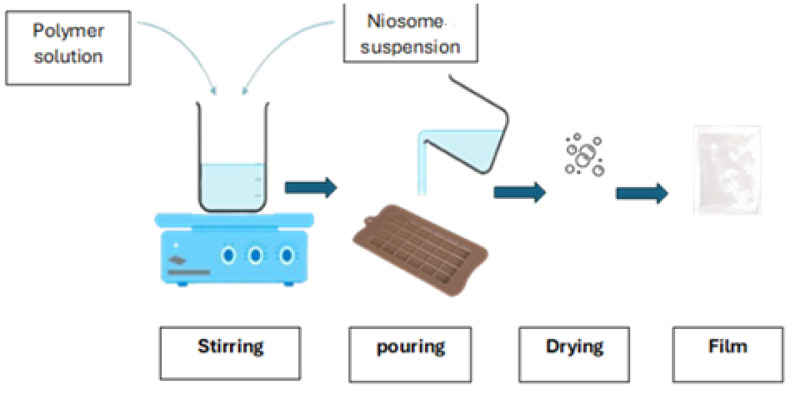
Solvent casting method for fast-disintegrating oral film.

The solvent casting method was used to prepare fast-disintegrating oral films; a film-forming matrix was first prepared. The selected polymer, polyvinyl alcohol (PVA), was dissolved in water 5% (*w/w*). A plasticiser, like polyethylene glycol 400 (1% *w/w*), was added to prevent brittleness (Table 2). Five millilitres of the prepared niosomal suspension was then incorporated into the polymer solution with gentle stirring to ensure uniform distribution. Sweeteners and flavouring agents, 0.2% *w/w*, were added to improve palatability. Finally, 1 mL of the solution was poured into each cubic space of the silicon mould and spread evenly to achieve a controlled film thickness (typically 100–200 µm). Different PVA concentrations (3%, 4%, 5%, and 6%) were evaluated in order to achieve the best mechanical characteristics and film flexibility. The results showed that films with less than 5% PVA were excessively brittle, whereas those with more than 5% PVA dissolved poorly. The 5% PVA formulation provided the optimum balance, combining strong mechanical qualities with an optimal dissolution time. The film was dried under a fume hood for 24 h until the solvent evaporated (Figure 5). Once it dried, the film was removed, typically with a dimension of 3 × 2 cm containing 0.5 mg of niosomal vancomycin for a single dose, and stored in a dry environment, protected from light and moisture.

#### 2.2.8. Characterisation of Fast-Disintegrating Oral Films

The weight variation was assessed using an analytical balance across the batches, with the average weight and standard deviation calculated to ensure consistency. Film thickness was measured at four distinct points using a Micrometre Screw clipper (Philip Harris, Accrington, UK) to confirm uniform drying and structure. The hand folding test was conducted by counting the number of folds before cracking, determining flexibility. Tensile strength (Chatillon cs2+ digital force tester, Berwyn, PA, USA) was measured with a texture analyser, recording the stress at the breaking point to assess film resilience. Film surface and dissolving solution pH values were tested by immersing the films in water and using Simplex Health universal pH test strips to ensure compatibility with the oral cavity, maintaining a neutral pH to prevent irritation. Finally, the in vitro disintegration time was measured by submerging film strips in 10 mL deionised water at 37 °C to record the time taken to disintegrate.

## 3. Results and Discussion

### 3.1. The Effect of Using Different Non-Ionic Surfactant Concentrations

The outcomes of utilising the microfluidic method to form nanoparticulate systems demonstrate the effects of varying total surfactant concentrations and aqueous-to-organic phase ratios with a constant vancomycin concentration (1 mg/mL) on the vesicle size, polydispersity index (PDI), and encapsulation efficiency (EE%).

The data in Table 3 show that increasing the total non-ionic surfactant concentration from 20 mg/mL to 60 mg/mL generally results in larger vesicle sizes, likely due to the greater availability of surfactant molecules to form larger or more aggregated structures. Additionally, a higher organic phase ratio (3:2) leads to larger vesicles when compared to a lower ratio (3:1), suggesting that the increased organic solvent content enhances the aggregation and fusion of surfactant molecules during nanoparticle formation. This was consistent with [31], where the change in the FRR from 1:1 to 3:1 for the same niosome formulation resulted in a decrease in vesicle size and distribution.

Regarding the PDI, which measures the uniformity of vesicle size, lower values indicate a more uniform size distribution. The data suggest that increasing the non-ionic surfactant concentration tends to decrease the PDI, especially at the 3:1 aqueous-to-organic ratio, implying that higher surfactant concentrations favour more uniform vesicle formation under these conditions. However, the PDI is generally higher at the 3:2 ratio, indicating a broader size distribution, potentially due to less controlled mixing or phase separation in a more organic environment.

The encapsulation efficiency (EE%) tends to increase with the increase in the surfactant concentration, particularly at the 3:1 aqueous-to-organic phase ratio, likely because a higher non-ionic surfactant amount provides more materials to encapsulate the active compound. However, at the 3:2 aqueous-to-organic phase ratio, the EE% is the highest at the lowest surfactant concentration (20 mg/mL) and decreases as the surfactant concentration increases. This may be due to increased viscosity and potential niosome aggregation at higher concentrations in a mixed solvent environment, which can reduce the encapsulation efficiency.

In conclusion, the microfluidic method allows for the fine-tuning of nanoparticle properties by adjusting the formulation parameters such as the non-ionic surfactant concentration and aqueous-to-organic phase ratio. These parameters affect the vesicle size, size distribution, and encapsulation efficiency, all of which are critical for the effectiveness and stability of nanoparticle-based drug delivery systems. Therefore, achieving optimal nanoparticle formulations requires balancing these factors according to specific application requirements.

### 3.2. The Effect of Using Different Ratios of Non-Ionic Surfactants

The comparison between the two niosome formulations (Table 3 and Table 4) reveals a major difference in the vesicle diameter, polydispersity index (PDI), and encapsulation efficiency (EE%) based on variations in the non-ionic surfactant concentrations, aqueous-to-organic phase ratios, and specific ratios of cholesterol–Span 60–Kolliphor RH40. In the first formulation, which uses a ratio of 3.5:4.5:2, the vesicle sizes generally increase with surfactant concentration, ranging from 58.48 nm at 20 mg/mL to 240 nm at the 3:2 aqueous-to-organic ratio, indicating moderate size changes. This formulation also shows a decrease in the PDI with an increasing non-ionic surfactant concentration, suggesting more uniform vesicle sizes at higher non-ionic surfactant concentrations, with PDI values decreasing from 0.408 at 20 mg/mL to 0.118 at 60 mg/mL. The encapsulation efficiency in this formulation increases with an increasing non-ionic surfactant concentration, reaching a maximum of 50.16% at 60 mg/mL at the 3:1 ratio, although it slightly decreases at higher organic phase ratio (Table 3). In contrast, the second formulation (Table 4), which uses a ratio of 5:4:1, M2, produces larger vesicle sizes, especially at higher organic phase ratios. For example, the vesicle size at 20 mg/mL jumps to 1696 nm at the aqueous-to-organic phase ratio of 3:2, indicating that this surfactant composition and the increased cholesterol content led to much larger vesicles. The PDI in the second formulation varies widely, with some extremely low values (e.g., 0.032 at 40 mg/mL and 3:2 ratio) indicating very uniform vesicles but also higher values in other conditions, reflecting a less consistent size distribution. Notably, the second formulation shows a higher encapsulation efficiency, particularly at the 3:2 ratio, achieving up to 60.36% at 20 mg/mL and 60.59% at 60 mg/mL. This suggests that the larger vesicles formed in this formulation allow for more drug encapsulation, making it more suitable for applications requiring higher drug loading capacities. Overall, the first formulation is more appropriate for applications needing smaller, more uniform vesicles, while the second formulation is better for applications that benefit from larger vesicles and higher encapsulation efficiencies [32].

### 3.3. Effect of Using Different Vancomycin Hydrochloride Concentrations

The comparison between the two microfluidic formulations highlights how varying drug concentrations and aqueous-to-organic ratios affect the properties of niosomes, specifically their size, polydispersity index (PDI), and encapsulation efficiency (EE%). In the first formulation (Table 5), using a 3.5:4.5:2 ratio of cholesterol, Span 60, and Kolliphor RH40, increasing the drug concentration from 0.5 mg/mL to 2 mg/mL with a 20 mg/mL surfactant concentration at the aqueous-to-organic phase ratio of 3:1 in all formulations shows an inconsistent trend in particle size, with sizes ranging from 120 nm to 130 nm. At the 3:2 ratio, the particle sizes are generally larger, indicating that a higher organic phase may lead to increased particle size, with the size at 2 mg/mL reaching 187.3 nm. The PDI values remain relatively low across all conditions, suggesting a fairly uniform size distribution, but there is a slight increase in the PDI when the drug concentration is increased to 2 mg/mL, which might indicate more variability in particle size. The encapsulation efficiency (EE%) in this formulation increases with the drug concentration, particularly at the 3:2 ratio, where it reaches 49.1% at 2 mg/mL, suggesting that larger particles formed at higher drug concentrations can encapsulate more drug. In contrast, the second formulation (Table 6), which uses a 5:4:1 ratio of cholesterol, Span 60, and Kolliphor RH40, displays different trends. At the 3:1 ratio, the particle size remains relatively stable across varying drug concentrations, around 138 nm. However, at the 3:2 ratio, there is more variability, with particle sizes ranging from 139.9 nm at 0.5 mg/mL to 191 nm at 1 mg/mL and then decreasing to 152.8 nm at 2 mg/mL. The PDI values in this formulation are generally lower at the 3:2 ratio, indicating a narrower size distribution, particularly at lower drug concentrations, where the PDI is as low as 0.055 at 0.5 mg/mL. This suggests that this formulation might provide more uniform particles under these conditions. The encapsulation efficiency shows a different pattern; it is the highest at the 3:2 ratio and the lowest drug concentration (0.5 mg/mL) at 78.03% but decreases as the drug concentration increases to 2 mg/mL. This trend may indicate that while this formulation can efficiently encapsulate lower drug concentrations, the efficiency drops as the concentration increases, possibly due to saturation effects or less optimal encapsulation conditions.

Overall, these comparisons demonstrate that the two formulations respond differently to changes in the drug concentration and aqueous-to-organic phase ratios, affecting the nanoparticle size, uniformity, and encapsulation efficiency. The first formulation (M1) seems to favour larger particles and higher encapsulation efficiencies at higher drug concentrations, while the second formulation (M2) is more efficient at encapsulating lower drug concentrations with a narrower size distribution. This highlights the importance of optimising the formulation parameters for desired nanoparticle characteristics in drug delivery applications.

### 3.4. Effect of Using Different Non-Ionic Co-Surfactants

The data from Table 7 illustrate the effects of using different non-ionic surfactants and co-surfactants with a constant vancomycin concentration (1 mg/mL) and constant surfactant concentration (20 mg/mL) on the properties of vesicles. Three niosomal formulation compositions were tested: cholesterol with Span 40 and Kolliphor ELP (M3 Ch-SP40-ELP,), cholesterol with Span 60 and Kolliphor ELP (M4 Ch-SP60-ELP), and cholesterol with Span 40 and Kolliphor RH40 (M5 Ch-SP40-RH40), all at a ratio of 3.5:4.5:2.

For M3 Ch-SP40-ELP formulation, the particle size increased from 188.3 nm at a 3:1 aqueous-to-organic phase ratio to 367.1 nm at a 3:2 ratio, indicating that the higher organic content promotes the formation of larger particles. The PDI values also increased slightly with the higher organic phase ratio, from 0.170 to 0.238, suggesting a broader size distribution at the 3:2 ratio. The encapsulation efficiency (EE%) improved significantly from 26.02% to 41.41%, aligning with the increase in particle size, which often correlates with higher encapsulation capacities.

In the M4 Ch-SP60-ELP formulation, the particle sizes were substantially larger than in the M3 Ch-SP40-ELP formulation, with a size of 420 nm at the 3:1 ratio and an exceptionally large size of 2159 nm at the 3:2 ratio. This size increase at the 3:2 ratio was accompanied by a very high PDI of 0.941, indicating a very broad size distribution and suggesting that particle formation was less controlled, possibly leading to aggregation. The encapsulation efficiency also increased with the higher organic phase, from 41.17% to 64%, suggesting that the large particles could encapsulate more of the active compound, although at the cost of uniformity and stability. For the M3 Ch-SP40-RH40 formulation, the particle sizes were the smallest among the three formulations, with 170.4 nm at the 3:1 ratio and 358.1 nm at the 3:2 ratio. The PDI values were also the lowest, at 0.128 for the 3:1 ratio and 0.165 for the 3:2 ratio, indicating that this formulation produced the most uniform particles. The encapsulation efficiency showed a moderate increase from 25.45% to 42.51% as the organic phase increased, suggesting a balance between the particle size and encapsulation capacity. Overall, the choice of non-ionic surfactants and co-surfactants affects the properties of niosomes. The use of Span 60 with Kolliphor ELP results in much larger and less uniform particles, particularly at higher organic phase ratios, which can be beneficial for encapsulation efficiency but may compromise stability. In contrast, using Span 40 with either Kolliphor ELP or Kolliphor RH40 results in smaller and more uniform particles, with moderate encapsulation efficiencies, indicating a more controlled nanoparticle formation process suitable for applications requiring stable and consistent nanoparticle sizes. The results are consistent with earlier findings where co-SAA types had an impact on the size and %EE (larger size and high EE when ELP was used) [33].

Studies on nanoparticle size for sublingual and buccal administration have primarily focused on particles between 100 and 300 nm. Investigations using porcine buccal mucosa have demonstrated that neutral polystyrene nanoparticles of varying sizes (25, 50, and 200 nm) can penetrate the tissue, with 200 nm particles showing the most efficient and deep penetration [34]. In contrast, smaller nanoparticles tend to be trapped within the mucous layer. Similarly, findings from human oral mucosa samples indicate that 200 nm nanoparticles can move beyond the epithelium and basement membrane into the connective tissue. However, since polystyrene nanoparticles are non-biodegradable and may interfere with normal cellular functions, further studies are required to explore biocompatible nanoparticle formulations across various size ranges [35]. This would provide valuable insights into their potential for enhancing drug absorption and mucosal permeability in sublingual and buccal drug delivery systems, refer to Section 3.7.

### 3.5. Niosome Drug Release Profile from Bag Dialysis

**Figure 6:**
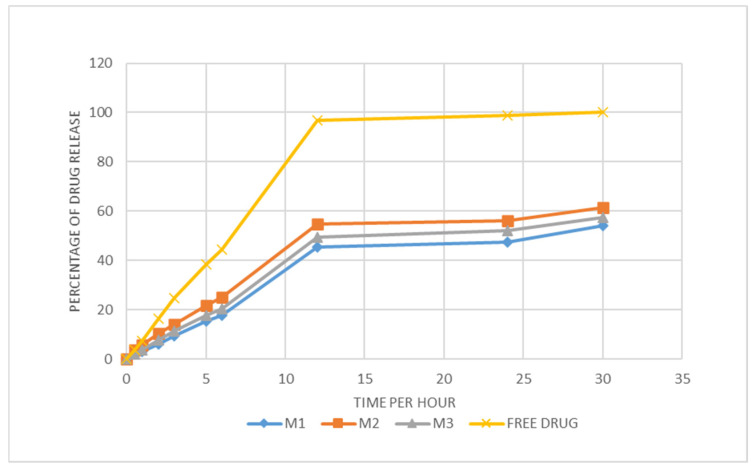
Release profile of vancomycin encapsulated within niosomes.

In the dissolution test for niosomes containing vancomycin, the most successful formulations in relation to the particle size, morphology, PDI, and EE% were used. Therefore, three different niosome formulations (M1, M2, M3), containing a constant vancomycin concentration (1 mg/mL) and constant surfactant concentration (20 mg/mL) in comparison to vancomycin alone (1 mg/mL) as the control, were evaluated for their drug release profiles over a 24 h period (Figure 6). Each formulation demonstrated similar results, releasing between 40% and 60% of their encapsulated cargo throughout the time duration. All formulations showed consistent and sustained drug release patterns, highlighting their potential for the delivery of vancomycin orally. Therefore, drug release pattern from niosomes is biphasic in nature, this was consistent with release profiles of insulin niosomes [14]. The formulations M1, M2, and M3 at a 3:2 microfluidic flow rate ratio reached 54.1%, 61.3%, and 57.3% release, respectively. The experiment was conducted for 24 h; accordingly, the same experiment could be conducted in the future, before in vivo studies, for a longer period of 72 h or until the complete release of the drug is achieved. However, less than 60% drug release from niosomes is expected within 24 h due to the composition of niosomes; our results are in accordance with data from [36,37].

Due to the high vancomycin concentration gradient between the niosomes inside the dialysis bag and the external buffer medium, along with the presence of the surface-adsorbed drug that diffuses out rapidly, the initial drug release was fast. Over time, the concentration gradient decreased, reducing the driving force for diffusion and slowing the release rate. Unlike systems that rely on erosion or dissolution, niosomal drug release is primarily governed by passive diffusion through the bilayer and dialysis membrane. Experiment conducted on atenolol niosomes showed that the release of drug from the niosomes fitted well to the Korsmeyer-Peppas kinetic model and drug release from different formulations were independent of the non-ionic surfactant type or ratio of cholesterol used in the niosomal formulation, the n values from these niosomes were <0.5, indicating drug release occurs via Fickian diffusion mechanism [38]. Regarding the free-drug release, the release profile showed a slow drug release in the first 6 h, where only about 40% of the drug was released; however, this slow drug release is due to using a dialysis bag with the free-drug release in this research (the normal USP dissolution tester has not been applied in this study, as this is not feasible for niosomes); hence, for fair comparison, the release of VCM as a free drug and as an encapsulated drug in niosomes was investigated using a dialysis bag, as detailed in Section 2.2.5. Accordingly, the free-drug release profile exhibited about 100% release within 12 h, which is a longer period.

The formula with the highest drug release is M2, which achieved the highest encapsulation efficiency (EE%) with an aqueous to organic phase ratio of 3:2; this indicates that better vancomycin release is proportional to the EE%. When a high entrapment efficiency is achieved, a larger proportion of the drug is retained within the carrier. This typically results in a more sustained and controlled release, as the drug is gradually released over time. For instance, a study on gliclazide-loaded niosomes demonstrated that formulations with higher entrapment efficiencies exhibited prolonged drug release over 24 h [36]. Also, the release of water-soluble peptides such as insulin niosomes has demonstrated a biphasic release pattern, the current data was consistent with release profiles of insulin nisosomes from previous works [14]. This suggests that the M2 formulation may offer the best performance in both drug release and the encapsulation efficiency, and it is a promising candidate for effectively delivering the antibiotic, showcasing its reliability in niosomal drug delivery systems.

### 3.6. Antimicrobial Test

**Figure 7:**
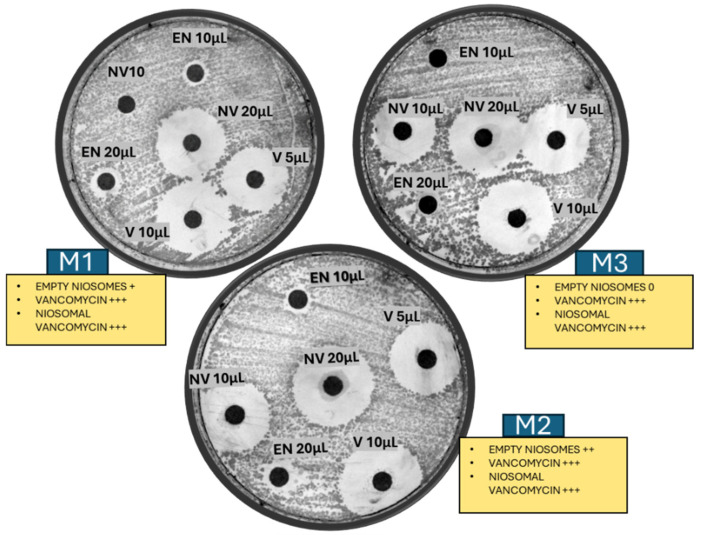
Agar diffusion test of vancomycin-loaded niosomes against *Bacillus subtilis*. EN, NV, and V refer to empty niosomes, niosomal vancomycin, and vancomycin alone, respectively. Symbols represent inhibition levels: +++ (strong, large zone), ++ (moderate, medium zone), + (weak, small zone), and 0 (no inhibition).

Nanodrugs can be more effective than traditional therapies at treating intracellular infections brought on by bacteria, viruses, fungi, or parasites [39]. In this study, we investigated the antibacterial efficacy of niosomes against *Bacillus subtilis* using the agar diffusion method. Empty niosomes produced inhibition zones of 8–11 mm, while vancomycin-loaded niosomes showed good activity with inhibition zones of 19–25 mm, similar to vancomycin alone (Figure 7), while there was no activity shown for the Gram-negative bacteria *Escherichia coli*. This demonstrates the effective encapsulation and release capabilities of niosomal formulations, highlighting their potential as a promising approach for combating bacteria. In order to maximise drug delivery by guaranteeing sustained release and targeted action, reducing adverse effects, and enhancing drug stability, nanoencapsulation techniques are being researched. Nanoencapsulation techniques are determined by the drug’s physicochemical characteristics. One important factor is the drug’s water solubility because water directly contributes to the creation of the exterior continuous phase of nanoparticles. The targeted delivery of antibiotics through niosomes also allows for higher local concentrations of the drug at the site of infection, leading to more effective bacterial eradication [19].

The adaptability and versatility of niosomes as a drug delivery system, coupled with their capacity for customisation to meet specific therapeutic requirements, underscore their substantial potential as a promising platform for advancing the efficacy and safety of various medical treatments, including the targeted killing of bacterial pathogens. Nanoparticles tagged with antibiotics further improve treatment by increasing the antibiotic concentration at the site of infection and strengthening antibiotic binding to bacteria [37,40].

### 3.7. Characterisation of Vancomycin-Loaded Niosome Fast-Disintegrating Oral Films (FDOFs)

The vancomycin-loaded niosome fast-dissolving oral films (FDOFs) were characterised based on key parameters to ensure quality and performance. The weight of the films, measured across the batch using an analytical balance, was on average 160 ± 2 mg, with a low standard deviation, confirming film-to-film consistency and uniformity. Film thickness, measured at four distinct points using a micrometre calliper, averaged 0.2 ± 0.0033 mm, indicating uniform drying and structural integrity, essential for consistent drug delivery. The films demonstrated excellent flexibility, with folding values exceeding 100 folds without cracking, highlighting their mechanical strength and durability for handling and administration. The tensile strength, measured using a texture analyser, revealed a stress at breaking point of 9.63 MPa. In the context of oral films, this value suggests that the films have a moderate to high degree of strength before failure, which is important for their durability during handling and packaging, confirming the films’ flexibility and ability to withstand mechanical stress. The surface pH of the films, tested by immersion in water and measured with pH strips, was maintained at close to neutral with pH 6.83 ± 0.4 for the film solution, ensuring compatibility with the oral cavity and minimising the risk of irritation. Additionally, regarding vancomycin disintegration, it was evaluated by submerging the films in 10 mL deionised water, the disintegration time was 105 s ± 12 s; this ensures rapid niosome release and, hence, is an indication of effective oral delivery. Overall, the vancomycin-loaded niosome FDOFs exhibited suitable uniformity, mechanical strength, flexibility, and rapid disintegration properties, confirming their suitability for oral delivery and ensuring both patient comfort and effective drug release.

## 4. Conclusions and Future Work

The development and characterisation of vancomycin-loaded niosome fast-dissolving oral films (FDOFs) and nanoparticle formulations demonstrate meaningful advancements in drug delivery systems. The microfluidic method enabled precise control over nanoparticle properties, with varying surfactant concentrations, aqueous-to-organic phase ratios, and surfactant compositions influencing the particle size, polydispersity index (PDI), and encapsulation efficiency (EE%). Formulations with higher surfactant concentrations and specific surfactant combinations, such as Span 40 with Kolliphor RH40, produced smaller, more uniform vesicles with moderate encapsulation efficiencies, while formulations with Span 60 and Kolliphor ELP resulted in larger particles with higher encapsulation capacities but broader size distributions. The vancomycin-loaded niosomes exhibited sustained drug release profiles, releasing 40–60% of the drug over 24 h, and demonstrated antibacterial efficacy against *Bacillus subtilis*, with inhibition zones comparable to those of free vancomycin.

The FDOFs exhibited excellent mechanical properties, including high folding endurance (>100 folds), tensile strength (9.63 MPa), and rapid disintegration (105 s), ensuring durability and patient compliance.

Overall, the vancomycin-loaded niosome FDOFs and nanoparticle formulations represent a promising approach for oral drug delivery, combining controlled release, enhanced stability, and effective antimicrobial activity. These findings highlight the potential of niosomal systems for improving the bioavailability and therapeutic efficacy of vancomycin, offering a viable alternative to conventional delivery methods. Further optimisation and clinical studies are warranted to fully realise their potential in therapeutic applications.

Regarding points to be considered for future work, the present work suggested the potential of the novel VCM niosome nanoformulation to eradicate *Bacillus subtilis*, which was used in this research as a model for performing the antimicrobial testing because it has well-studied resistance mechanisms and a structural resemblance to clinically applicable Gram-positive bacteria. Accordingly, testing of this novel formula on other clinically relevant pathogenic bacteria is needed in the future to confirm the effectiveness of the innovative VCM niosomes against a wide range of clinically relevant pathogens. Also, there may be adverse effects of the niosomes on the oral mucosa; therefore, cytotoxicity tests on oral epithelial cells are required in future testing to assess niosome biocompatibility before in vivo testing and the application of the oral fast-disintegrating VCM niosome films in clinical settings/studies.

## Figures and Tables

**Table 1:** Non-ionic surfactant composition of niosomal formulations (amounts as molar ratios).

Formulation	Niosome Composition	Cholesterol	Span 60	Span 40	Kolliphor RH40	Kolliphor ELP
M1	Ch-SP60-RH40	35%	45%		20%	
M2	Ch-SP60-RH40	50%	40%		10%	
M3	Ch-SP40-ELP	35%		45%		20%
M4	Ch-SP60-ELP	35%	45%			20%
M5	Ch-SP40-RH40	35%		45%	20%	

**Table 2:** Fast-disintegrating oral film ingredients.

Ingredient	Percentage (*w/v*)	Total Weight for 10 Films (mg)
Film-forming polymer (PVA)	5%	500 mg
Plasticiser (polyethylene glycol 400 (PEG 400))	1%	100 mg
Niosomal suspension	3.6%	360 mg
Sweetener (Sucralose)	0.2%	20 mg
Flavouring agent (Mint)	0.2%	20 mg

**Table 3:** Different non-ionic surfactant (organic phase) concentrations for M1 Formulation (cholesterol–Span 60–Kolliphor RH40 (3.5:4.5:2)) at 1 mg/mL initial VCM loading.

Total Non-Ionic Surfactant (Organic Phase) Concentration	Aqueous-to-Organic Phase Ratio	Size (nm)	PDI	EE%
20 mg/mL	3:1	58.48 ± 0.676	0.408 ± 0.002	24.89
	3:2	240 ± 1.5	0.240 ± 0.013	49.09
40 mg/mL	3:1	90.29 ± 0.78	0.132 ± 0.013	30.17
	3:2	191.5 ± 2	0.202 ± 0.014	43.55
60 mg/mL	3:1	133.6 ± 1.51	0.118 ± 0.009	50.16
	3:2	210.1 ± 4.35	0.215 ± 0.013	42.18

**Table 4:** Different non-ionic surfactant (organic phase) concentrations for M2 Formulation, cholesterol–Span 60–Kolliphor RH40 (5:4:1), at 1 mg/mL VCM loading.

Total Non-Ionic Surfactant Concentration	Aqueous-to-Organic Ratio	Size (nm)	PDI	EE%
20 mg/mL	3:1	142.1 ± 2.7	0.142 ± 0.029	30.52
	3:2	1696 ± 17	0.32 ± 0.004	60.35
40 mg/mL	3:1	112.1 ± 0.85	0.179 ± 0.005	32.15
	3:2	137.3 ± 0.72	0.032 ± 0.024	43.12
60 mg/mL	3:1	136 ± 1.22	0.135 ± 0.008	24.44
	3:2	1082 ± 40.3	0.188 ± 0.11	60.59

**Table 5:** Different vancomycin (aqueous phase) concentrations for M1 Formulation: cholesterol–Span 60–Kolliphor RH40 (3.5:4.5:2). Surfactant concentration was 20 mg/mL.

Drug Concentration	Aqueous-to-Organic Ratio	Size (nm)	PDI	EE%
0.5 mg/mL	3:1	130 ± 1.3	0.164 ± 0.03	26.09
	3:2	188.3 ± 3.15	0.155 ± 0.017	43.85
1 mg/mL	3:1	137.6 ± 1	0.156 ± 0.026	28.70
	3:2	155.5 ± 2.1	0.151 ± 0.017	41.35
2 mg/mL	3:1	120 ± 0.6	0.147 ± 0.01	34.18
	3:2	187.3 ± 1	0.181 ± 0.014	49.10

**Table 6:** Different vancomycin (aqueous phase) concentrations for M2 Formulation: cholesterol–Span 60–Kolliphor RH40 (5:4:1). Surfactant concentration was 20 mg/mL.

Drug Concentration	Aqueous-to-Organic Ratio	Size (nm)	PDI	EE%
0.5 mg/mL	3:1	138.2 ± 1.4	0.158 ± 0.018	36.89
	3:2	139.9 ± 0.7	0.055 ± 0.014	78.03
1 mg/mL	3:1	132 ± 1.4	0.181 ± 0.003	35.78
	3:2	191 ± 19.8	0.338 ± 0.082	45.03
2 mg/mL	3:1	139 ± 1.2	0.112 ± 0.009	21.48
	3:2	152.8 ± 2.5	0.090 ± 0.014	43.16

**Table 7:** Different types of non-ionic surfactant (organic phase) components for Formulations M3, M4, and M5 at a ratio of 3.5:4.5:2, 1 mg/mL drug loading, and 20 mg/mL surfactant concentration.

Non-Ionic Surfactant Composition	Aqueous-to-Organic Ratio	Size (nm)	PDI	EE%
M3 Ch-SP40-ELP	3:1	188.3 ± 3.33	0.170 ± 0.006	26.01
	3:2	367.1 ± 3.06	0.238 ± 0.021	41.41
M4 Ch-SP60-ELP	3:1	420 ± 4.86	0.292 ± 0.041	41.17
	3:2	2159 ± 322.2	0.941 ± 0.103	63.99
M5 Ch-SP40-RH40	3:1	170.4 ± 1.40	0.128 ± 0.021	25.44
	3:2	358.1 ± 4.65	0.165 ± 0.008	42.51

## Data Availability

Data is contained within the article.
